# Studies on the Complexation of Platinum(II) by Some 4-Nitroisoxazoles and Testing the Cytotoxic Activity of the Resulting Complexes

**DOI:** 10.3390/molecules28031284

**Published:** 2023-01-28

**Authors:** Henryk Mastalarz, Agnieszka Mastalarz, Joanna Wietrzyk, Magdalena Milczarek, Andrzej Kochel, Andrzej Regiec

**Affiliations:** 1 Department of Organic Chemistry and Drug Technology, Faculty of Pharmacy, Wrocław Medical University, 211A Borowska Street, 50-556 Wrocław, Poland; 2 Faculty of Chemistry, The University of Wrocław, 14F Joliot-Curie Street, 50-383 Wrocław, Poland; 3 Hirszfeld Institute of Immunology and Experimental Therapy, Polish Academy of Sciences, 12 Rudolf Weigl Street, 53-114 Wrocław, Poland

**Keywords:** 4-nitroisoxazoles-Pt(II) complexes, synthesis, structural analysis, LogP, cellular platinum uptake, cytotoxic activity, normoxia, hypoxia, L-glutathione (GSH), X-ray crystallography

## Abstract

Two novel platinum(II) complexes (**1** and **2**) were synthesized by the reaction of the appropriate 3,5-dimethyl-4-nitroisoxazole with K_2_PtCl_4_ and characterized by elemental analysis, ESI MS spectrometry, ^1^H NMR and far-IR spectroscopy. The structure of *trans* complex **2** was additionally confirmed by X-ray diffraction. The cytotoxicity of the investigated compounds was examined in vitro on three human cancer cell lines (MCF-7 breast, ES-2 ovarian and A-549 lung adenocarcinomas) in both normoxia and hypoxia conditions. LogPs of complexes were measured using the shake-flask method. The *trans* complex **2** showed much better cytotoxic activity than cisplatin for all the tested cancer cell lines. *Cis* complex **1** was inferior to its *trans* isomer against all the cancer lines tested in normoxia conditions but proved superior to the reference cisplatin against the MCF-7 and A549 lines, and showed similar activity to cisplatin against the ES-2 line. To gain additional information that may facilitate the explanation of the pharmacological activity of the tested compounds, cellular platinum uptake and stability in L-glutathione solution were determined for both compounds **1** and **2**.

## 1. Introduction

Chemotherapy is currently a commonly used and effective method of cancer treatment [1]. Its beginnings date back to the early forties when the ability to inhibit the process of cell division was discovered as a result of the action of mustard gas derivatives. Until the late 1960s, all drugs used to treat cancer were organic compounds, including folic acid antagonists, purine antimetabolites, and vinca alkaloids [1,2]. In the late 1960s, the cytostatic properties of a simple coordination compound called cisplatin were accidentally discovered on the basis of its ability to inhibit bacterial growth. This discovery opened new possibilities in the search for new drugs that were effective in cancer chemotherapy [3]. Platinum-derived anticancer drugs, such as cisplatinum, carboplatinum and oxaliplatinum, with clear therapeutic effects and a well-studied mechanism of action, are now widely used in clinical practice [4]. At the same time, new platinum drugs with a non-classical structure are also still being sought [5]. However, platinum(II) derivatives currently used in medicine are non-specific drugs, causing many undesirable side effects and showing significant systemic toxicity [6]. The adverse features of currently used platinum drugs include, in particular, dose-limiting general toxicity, nephrotoxicity, neurotoxicity, ototoxicity and myelosuppression [6,7,8]. In addition, long-term use of cisplatin has been found to cause serious damage to normal body tissues [7]. The observed significant therapeutic effect of cisplatinum and other first-line platinum drugs on the development of a tissue tumor means that there is still interest in the design and synthesis of this class of compounds with reduced systemic toxicity and maximum activity in cancer treatment. Likewise, there is also interest in the possibility of activating the platinum drug only when it reaches the cancerous tumor directly. Increasing biosafety while maintaining anticancer activity would create even better prospects for these drugs in the treatment of cancer [4]. One strategy that would avoid these unwanted side effects is to use new complexes with different mechanisms of action. Many studies on inorganic compounds focused on the search for new metal complexes apart from platinum, such as copper, gallium, ruthenium, cobalt, reduced palladium and gold complexes, but they have not yet brought the expected results [9,10,11]. Nevertheless, the search for new drugs based on Pt is still a current problem due to the potential of this class of compounds as potent anticancer agents, which has recently led some of them to be tested in preclinical studies [12,13,14,15]. Advances in research on platinum-based anticancer drugs describing a different approach, including non-classical structures, have been announced in numerous publications [16,17,18,19]. However, despite efforts in this area, no new platinum complex drug has been introduced into anticancer therapy for more than 10 years [20,21]. In our previous studies [22], we synthesized and characterized a series of *cis* and *trans* platinum(II) complexes with the structural formula trans-[PtCl_2_L_2_] with substituted nitropyrazole ligands. Cytotoxicity evaluation on a panel of three tumor cell lines revealed a significant potential of trans-[PtCl_2_ (1-methyl-4-nitropyrazole)_2_], which exhibited cytostatic activity in the micromolar concentration range, with the highest cytotoxicity against ES-2 cells, surpassing that of cisplatinum, while the appropriate *cis* congeners showed only moderate activity. An attempt was also made to elucidate the molecular mechanisms underlying the in vitro biological activity of trans [PtCl_2_ (1-methyl-4-nitropyrazole)] by examining its effect on the cell cycle, and we evaluated the mechanism of cytotoxic action as a result of the ability of the complex to bind intracellular DNA and proteins. We also conducted a study of the reactivity of the obtained compounds with glutathione in order to assess the rate of their deactivation in living organisms. This work is a continuation of this topic, but now we have directed our interests to the area of 4-nitroisoxazole derivatives as potential ligands. This choice was related to our previous studies, which strongly suggested that the cytotoxicity of nitroazole complexes with Pt(II) requires the presence of a heteroatom–heteroatom bond in the ligand molecule used. The anticancer properties of Pt(II) complexes with nitroisoxazoles have never been investigated so far. However, a number of methods for the synthesis of these types of nitroisoxazole ligands are known, including the direct nitration of isoxazoles in the HNO_3_-H_2_SO_4_ system [23] or acyl nitrates [24], NO/NO_2_-mediated heterocyclization of α,β-unsaturated ketones [25], the reaction between N-hydroxylamine and β-nitroenamines with a formyl group at the β-position [26] or 1,4-dinitropyrazole [27].

## 2. Results and Discussion

### 2.1. Synthesis and Structural Analysis of Pt-Complexes

**Figure 1:**
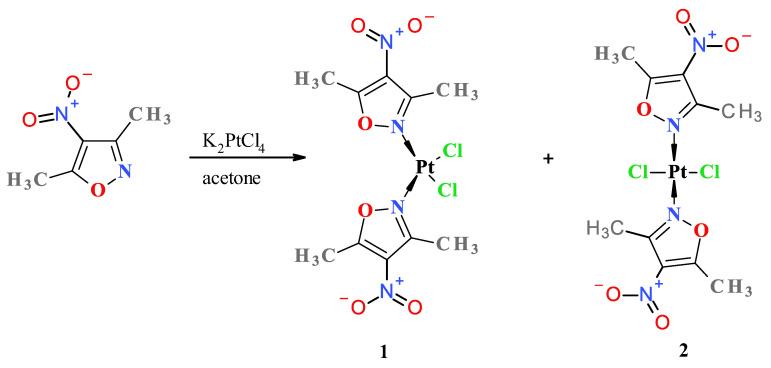
Scheme of synthesis of platinum(II) complexes **1** and **2**.

To obtain the desired nitroisoxazole complexes, we synthesized four known 4-nitroisoxazole derivatives according to the previously described procedures, namely, 3-methyl-4-nitroisoxazole [23], 4-nitroisoxazole, 5-methyl-4-nitroisoxazole and 3,5-dimethyl-4-nitroisoxazole [24]. Complexation reactions were carried out in darkness at room temperature by mixing an acetone solution of two (2) molar equivalent of an appropriate ligand with a water solution of potassium tetrachloroplatinate (Figure 1). Reaction times were established by using TLC chromatography. Further workup of reaction mixtures depended on the nature of the formed products.

Considering the unsatisfactory yield (10%) of the more antitumor-active (relative to *cis*-isomer 1) *trans* complex **2** during the standard procedure, and based on the previous results obtained during the synthesis and study of the properties of complexes with nitropirazoles, for which it was possible to convert the platinum *cis* complex with 1-methyl-4-nitropyrazole into its *trans* isomer very easily and with high efficiency by thermal isomerization [22], we decided to convert *cis* Pt(3,5-dimethyl-4-nitroisoxazole) complex **1** into its *trans* isomer. It turned out, however, that in this case, such isomerization does not take place, because the complete thermal decomposition of complex **1** takes place. However, on further investigation, we found that upon prolonged storage of compound **1** in dioxane solution, it slowly isomerizes to the mixture containing its *trans* congener, allowing an additional amount of compound **2** to be obtained for biological study.

**Figure 2:**
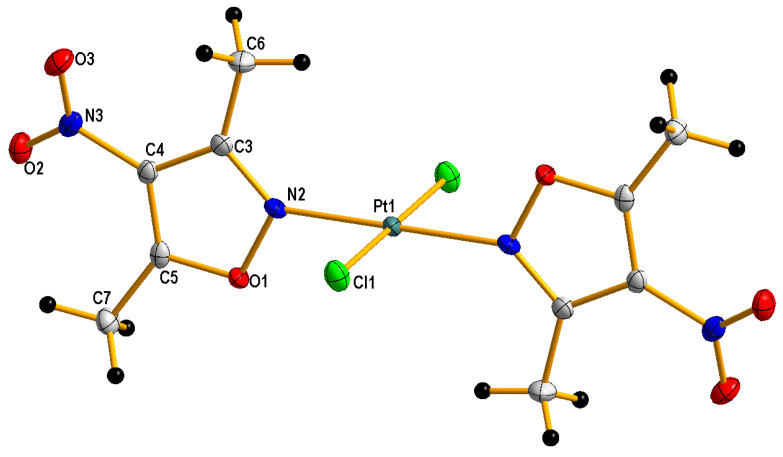
Crystal structure of trans-dichlorobis(3,5-dimethyl-4-nitro-isoxazole)platinum(II) with displacement ellipsoids drawn at the 50% probability level. Labelling scheme for asymmetric unit.

Additionally, it is worth adding that complexation with 4-nitroisoxazole 3-methyl-4-nitroisoxazole and 5-methyl-4-nitroisoxazole was unsuccessful probably due to the decomposition of ligands in the reaction environment. Both novel platinum(II) complexes **1** and **2** were characterized by elemental analysis, ^1^H NMR spectroscopy (the visualizations of the ^1^H NMR spectra, see Figures S7 and S8 in the Supplementary Materials) and far-IR spectroscopy (for the visualizations of the far-IR spectra, see Figures S4 and S5 in Supplementary Materials) and ESI mass spectrometry (for the visualizations of the MS-simulated and experimental spectra, see Figures S1–S3 in the Supplementary Materials). Furthermore, compound **2** was analyzed by X-ray diffraction (XRD) (Figure 2 and crystallographic data collected in Tables S1–S7 in the Supplementary Materials). Far-IR spectroscopy is very useful for distinguishing *cis* from *trans* isomers for platinum complexes. *Cis* and *trans* congeners were distinguished through their far-IR spectra at the 400–300 cm^−1^ range because both *cis* and *trans* isomers exhibit a strong Pt-Cl stretching vibration (at about 340–360 cm^−1^ range), which is split into two bands only in the case of complexes with *cis* geometry. The reason is that both symmetric (ν_s_Cl-Pt = 355 cm^−1^ for *cis* complex **1**) and antisymmetric (ν_as_Cl-Pt = 347 cm^−1^ *cis* complex **1**) valence vibrations of *cis* Pt(II) complexes are active ones contrary to *trans* isomers, in the case of which only antisymmetric vibration (ν_as_Cl-Pt = 344 cm^−1^ for *trans* complex **2**) is active [28]. In the case of the ^1^H-NMR spectroscopy of both complexes (**1** and **2**) and their ligand (3,5-dimethyl-4-nitroisxazole), the effect of electron deshielding of the methyl protons caused by electron withdrawal by the central platinum ion is clearly visible, which is in line with expectations. It can be seen that the protons of the methyl group at position 3 of the isoxazole ring are more strongly deshielded than the protons of the methyl group at position 5 due to its closer proximity to the platinum ion. This effect is stronger in the case of *trans* complex **2** than in the case of *cis* isomer **1** (see the values of chemical shifts in see chapter 3.2.2 of the experimental part and visualization of the spectra in Figures S6–S8 in the Supplementary Materials). However, it should be emphasized that the unambiguous way of distinguishing *cis* from *trans* isomers of platinum(II) complexes is far-IR and the single crystal X-ray diffraction (XRD) method.

### 2.2. Lipophilicity

As an important factor for the pharmacokinetics and cellular accumulation of the investigated complexes, the lipophilicity of each complex was determined by measuring octanol/water partition coefficients (log P) with the shake-flask method [29]. The log P determination was carried out based on a previously described procedure [30] modified by us to obtain more accurate and repeatable results. The results are collected in Table 1. Our measured log P value (−2.05) for cisplatin is within the range of previously reported values (−2.27 and −1.74) [29], which confirms the correctness of our methodology. Similarly to our previous results [22], the current findings also support the general rule that *trans* platinum complexes are more lipophilic (higher log P values) than their corresponding *cis* isomers, which may be related to their zero electric dipole moments because of their centrosymmetric molecules. Furthermore, as you can see (Table 1), both tested complexes **1** and **2** are more lipophilic than the reference cisplatin.

### 2.3. Cellular Platinum Uptake

The uptake of both Pt complexes and reference cisplatin by MCF-7 breast cancer cells was measured after 4 h exposure at 10 µM concentration according to the previously described protocol [31] using inductive coupled plasma spectroscopy (ICP OES) for platinum determination. The results are presented in Table 1. Statistical analysis of the data indicates that both complexes **1** and **2** were absorbed significantly less than cisplatin by MCF-7 cells, but there is no statistically significant difference in absorption between these two isomers (for Fisher LSD, Bonferroni, Tukey HSD and Dunnett’s and post hoc test, the threshold for significance level was set at α = 0.05. The results were considered statistically significant, when calculated probability of statistical significance had *p*-value < 0.05). Despite differences in lipophilicity between *cis* and *trans* isomers, no differences in the process of platinum absorption by MCF-7 cells were observed. Similar observations were made in the case of complexes with nitropyrazoles [22].

### 2.4. In Vitro Cytotoxic Activity

The cytotoxicity of the test compounds and the reference cisplatin was determined through a cell viability test with the sulforhodamine B assay and expressed as a 50% inhibitory concentration (IC_50_) which causes a 50% reduction in the cell viability.

The *trans* complex **2** showed much better cytotoxic activity than the reference drug cisplatin for all the tested cancer cell lines (Table 2). *Cis* complex **1** was inferior to its *trans* isomer **2** against all the cancer lines tested in normoxia conditions but proved superior to the reference cisplatin against the MCF-7 and A549 lines and showed similar activity to cisplatin against the ES-2 line. For *cis* complex **1**, there was no statistically significant difference in activity against all tumor lines between normoxia and hypoxia conditions. For the *trans* complex **2**, a statistically significant difference was found for activity against the cancerous MCF7 and ES2 lines between normoxia and hypoxia conditions. In these cases, *trans* complex **2** was found to be more active under normoxia conditions. The reference cisplatin showed a statistically significant difference in activity only for the A549 line, where it proved more active in normoxia.

Both complexes **1** and **2** are statistically less toxic for healthy cells than against the MCF-7 cell line. Cytotoxicity of cisplatin against healthy cells is statistically comparable to cytotoxicity against the MCF-7 cell line. So, the selectivity indexes (SI), i.e., the ratio of cytotoxicity against healthy cells to cancerous ones, for *cis* complex **1** and *trans* complex **2** are ≈2.0 and ≈3.0, respectively. SI for reference drug cisplatin is ≈1.

Both complexes **1** and **2** are statistically less toxic for healthy cells than against the ES-2 cell line. The cytotoxicity of cisplatin against healthy cells is statistically comparable to the cytotoxicity against the ES-2 cell line. So, the selectivity indexes (SI) for *cis* complex **1** and *trans* complex **2** are ≈1.4 and ≈1.7, respectively. The SI for the reference drug cisplatin is ≈1.

*Cis* complex **1** is statistically less toxic for healthy cells than against the A549 cell line. The cytoxicity of cisplatin against the healthy cells of both trans complex **2** and cisplatin is statistically comparable to the cytotoxicity against the A549 cell line. So, the selectivity index (SI) for *cis* complex **1** is ≈1.2. However, the SI for both *trans* complex **2** and the reference drug cisplatin is ≈1.

In conclusion, on the basis of the results of cytotoxicity studies conducted in vitro, it can be said in general that the *trans* complex shows significantly greater cytotoxic activity against all tested tumor lines in relation to both the *cis* isomer and the reference drug cisplatin. At the same time, the *trans* isomer shows significantly greater cytotoxicity against healthy cells than the *cis* isomer and reference cisplatin; however, it has a slightly better selectivity index (SI) than the *cis* isomer and cisplatin except in the case of tumor line A549, where toxicity against healthy and tumor cells is comparable. It should also be noted that, to the best of our knowledge, compound **2** is one of the most cytotoxic Pt(II) complexes reported in the pharmacological literature.

### 2.5. Reactivity with L-Glutathione (GSH)

**Figure 3:**
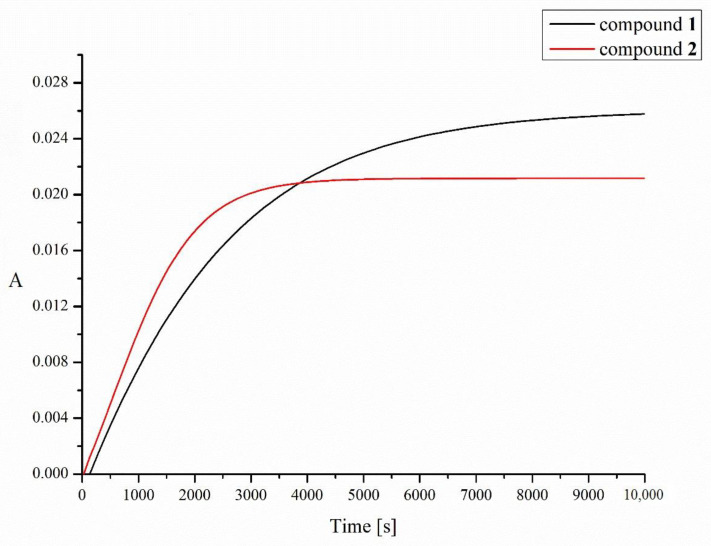
Time dependence of UV absorbance (at 260 nm) of compounds **1** (black line) and **2** (red line) in the presence of 2 mM of L-glutathione (GSH). A is absorbance.

Pt(II) complexes have a strong ability to react with sulfur donor ligands. Thus, before the platinum-containing molecule reaches the tumor tissue, it may be deactivated by reactions with various endogenous sulfur-containing molecules. These side reactions are believed to play an important role in the mechanisms of tumor resistance to platinum drugs, their inactivation and toxic side effects [5]. The two most important endogenous thiols to which platinum complexes can bind after administration or after entering a tumor cell are reduced forms of L-glutathione (GSH) and metallothionein (MT) [32]. In this work, we studied the reactions of GSH with compounds **1** and **2** by measuring the UV absorbance at 260 nm (indicating the formation of Pt-S and S-S bonds) as a function of time [33] using a double-beam spectrophotometer (Figure 3). Due to the insufficient solubility of our compounds in water, dioxane was used to prepare a stock solution of the tested compounds, which was then diluted with an appropriate amount of an aqueous solution of L-glutathione (GSH). Despite the better solubility of the tested complexes in acetone, dioxane was used in this experiment due to the lack of absorption of UV radiation at the wavelength of 260 nm, which is in contrast to acetone, which strongly absorbs at this wavelength. As shown in Figure 3, the increase in absorbance of the solution of complexes **1** and **2** and L-glutathione over time clearly indicates that a chemical reaction is taking place, resulting in an absorbance-enhancing product at the wavelength used.

The half-times of the reaction with 2 mM GSH, resulting mainly in the formation of Pt-S bonds, were found to be 42 and 18 min for compounds **1** and **2**, respectively. Increasing the concentration of L-glutathione to 4, 8 or 16 mM does not significantly affect the measurement results. For comparison, the half-life of cisplatin in glutathione solution under similar conditions was previously determined to be 53 min [32].

## 3. Experimental

The melting points (mp) of the compounds were measured on the Büchi M560 melting point device (BÜCHI Labortechnik AG, CH-9230 Flawil/SG, Flawil, Switzerland) and were uncorrected. Elemental analyses were performed by the Laboratory of Elemental Analyses, Faculty of Chemistry, Wrocław University, with a 2400 CHN elemental analyzer (Perkin-Elmer, Waltham, MA, USA). The ESI-MS (electrospray ionization mass spectroscopy) spectra were recorded with the compact^TM^ Bruker Daltonics Electrospray Ionization Quadrupole Time-of-Flight (ESI-Q-TOF) apparatus (Bruker Daltonics GmbH, Bremen, Germany). The ESI-MS experiments were carried out in negative-ion mode in LC–MS-grade methanol as a solvent. Bruker Compass Data Analysis 4.2 software (Bruker Daltonics GmbH, Bremen, Germany) was used for ESI-MS spectral analysis and the simulation of the theoretical monoisotopic mass of detected ions. The far-IR spectra (50–650 cm^−1^) were recorded with the Bruker 113v FTIR spectrophotometer (Bruker, Germany) in nujol mull. The ^1^H-NMR (300.15 MHz) spectra were recorded using a Bruker ARX-300 spectrometer (Bruker Analytische Messtechnik GmbH, Rheinstetten, Germany). The NMR measurement of samples of the compounds were carried out in deuterated acetone-d_6_. ^1^H-NMR chemical shifts were referenced to the solvent signal, i.e., for ^1^H-NMR: δ (quintet of residual acetone-d_6_) = 2.05 ppm. Platinum content in biological and logP samples was measured with the iCAP 7400 Duo ICP-OES analyzer (Thermo Fisher Scientific) running Thermo Scientific™ Qtegra™ Intelligent Scientific Data Solution™ (ISDS) integrated software, version 2.10. The thin-layer chromatography (TLC) method was used to monitor the course of the reaction and to determine the purity of the synthesized compounds. TLC-Al plates with a fluorescent indicator of 254 nm were used, with silica gel as the stationary phase (Fluka), and chloroform-acetone (volume ratio 9:1) or chloroform-methanol (volume ratio 95:5) was used as the eluent. The detection of compounds on chromatograms was carried out with a 0.5% acetone solution of rubeanoic acid (dithiooxamide) by observing the plate under UV light at 250 nm and/or developing the chromatogram with iodine vapor.

### 3.1. Synthesis of 4-Nitroisoxazole Ligands

All nitroisoxazole ligands were obtained by an electrophilic substitution reaction in the ring of the respective isoxazole derivative. 3-Methyl-4-nitroisoxazole was synthesized by the method described in [23] by direct nitration of 3-methylisoxazole in H_2_SO_4_-HNO_3_ mixture. The remaining ligands, such as 4-nitroisoxazole, 5-methyl-4-nitroisoxazole and 3,5-dimethyl-4-nitroisoxazole, were prepared according to the previously described methods through nitration in an anhydrous nitric acid/trifluroacetic anhydride system [24].

### 3.2. Synthesis of Platinum(II) Complexes

#### 3.2.1. General Description of the Synthesis of Complexes

Solutions of 2 mmol of appropriate organic ligands in 10 mL of acetone and 415 mg (1 mmol) of K_2_PtCl_4_ in 10 mL of water were combined and left in a sealed reaction flask protected from light for at least 1 month. After this time, the precipitate was filtered off and, in each case, except for 3,5-dimethyl-4-nitroisoxazole, it was found to be poorly soluble in common organic solvents, except DMF, and the chromatographic analysis (TLC) indicated that the precipitate was a multi-component mixture of products. It was not possible to isolate from none of the reaction mixtures an individual product that could be subjected to further research, except if 3,5-dimethyl-4-nitroisoxazole was used. Only two complexes (*cis* and *trans*) with 3,5-dimethyl-4-nitroisoxazole were successfully formed, the detailed description of the preparation and spectral parameters of which are given below.

#### 3.2.2. Complexation with 3,5-Dimethyl-4-nitroisoxazole Resulting in Cis and Trans Dichlorobis(3,5-Dimethyl-4-nitroisoxazole)Platinum(II)

Solutions of 284 mg (2 mmol) of 3,5-dimethyl-4-nitroisoxazole in 10 mL of acetone and 415 mg (1 mmol) of K_2_PtCl_4_ in 10 mL of water were combined and left in a sealed reaction flask protected from light. After two months, the solution changed color from dark red to light orange, and a precipitate crystallized at the bottom of the reaction vessel, which was filtered off and dried in vacuum. An amount of 280 mg (total yield 50.9%) of crude product was obtained, that was soluble in acetone, slightly soluble in chloroform, and poorly soluble in CCl_4_ and water. TLC showed it to be a mixture of two platinum-containing compounds. The mixture was separated by column chromatography on a 200–30 mesh SiO_2_ column. The mixture was initially applied as a chloroform solution and developed with chloroform, then the polarity of the eluent was increased gradually by the addition of acetone, starting from a 9:1 volume ratio and finally reaching a CHCl_3_: acetone (4:1 volume ratio) system. The result was two chromatographically homogeneous fractions. The more polar one contained *cis* isomer **1** and the less polar one contained *trans* isomer **2**. After evaporating the solvents from each fraction, each separated precipitate was independently crystallized from acetone. An amount of 135mg of yellow *cis* isomer **1** from the more polar fraction was obtained (yield 24.5%). The mp of *cis*-isomer **1** is 160 °C (decomposition). Complex **1** is weakly soluble in acetone, much weaker in chloroform and very weakly soluble in carbon tetrachloride and water. From a less polar fraction, 67 mg (12.2% yield) of the light-yellow *trans* complex **2** was separated. The mp of *trans* isomer **2** is 200 °C (decomposition). It is quite soluble in acetone, better than *cis* isomer **1**.

Combustion and spectral analyses for the *cis* complex **1:** Elemental analysis for formula C_10_H_12_N_4_O_6_Cl_2_Pt of compound **1** (calculated/found (%)): C 21.83/21.59, H 2.20/2.31, N 10.18/9.89, Cl 12.89/12.26. ^1^H NMR (300.15 MHz, acetone-d_6_): δ[ppm] 2.88 (s, 3H, CH_3_ in third position of isoxazole ring), 3.01 (s, 3H, CH_3_ in fifth position of isoxazole ring). Far-IR (nujol) ν[cm^−1^]: 355 (strong, ν_s_Cl-Pt), 347 (strong, ν_as_Cl-Pt). Visualizations of far-IR and ^1^HNMR spectra of compound **1** are in the Supplementary Materials (Figures S4 and S7, respectively).

Combustion and spectral analyses for the *trans* complex **2:** Elemental analysis for formula C_10_H_12_N_4_O_6_Cl_2_Pt of the *trans* complex **2** (calculated/found (%)): C 21.83/22.12, H 2.20/2.56, N 10.18/10.50, Cl 12.89/12.31. ^1^H NMR (300.15 MHz, acetone-d_6_): δ[ppm] 3.00 (s, 3H, CH_3_ in third position of isoxazole ring), 3.06 (s, 3H, CH_3_ in fifth position of isoxazole ring). Far-IR (nujol) ν[cm^−1^]: 344 (strong, ν_as_Pt-Cl). Visualizations of far-IR and ^1^HNMR spectra of compound **2** are in the Supplementary Materials (Figures S5 and S8, respectively).

Analysis of mass spectra (ESI-MS) of both complexes **1** and **2**: The calculated value of the parent peak mass for the formula C_10_H_12_N_4_O_6_Cl_2_Pt is 548.97817 u. The ESI-MS (negative ionization in methanol) of **1** and **2** revealed, amongst others, the following peaks given as a ratio m/z [u/e] (relative intensity to base peak [%]): 548.965197 (100%) [PtL_2_Cl_2_+1-H]^−^ —isotope peak of quasi-molecular ion (base peak), 547.967118 (73.7%) [PtL_2_Cl_2_-H]^−^—quasi-molecular ion (parent) peak, 405.929436 (31.8%) [PtLCl_2_-H]^−^—fragmentation ion peak, where L means ligand, i.e., 3,5-dimethyl-4-nitroisoxazole. Visualizations of ESI-MS spectra are in the Supplementary Materials (Figures S1–S3)

### 3.3. Single Crystal X-ray Structure Determination of Complexes

A crystal of *trans* complex **2** suitable for XRD measurement was obtained by slowly evaporating the solvent from its acetone solution. Crystallographic measurement for compound **2** was performed with Κ-geometry diffractometers: Xcalibur Ruby, Gemini ultra with graphite monochromatized Mo-Kα radiation (λ = 0.71073 Å) at 100(2) K, using an Oxford Cryosystems cooler. Data collection, cell refinement, data reduction and analysis were conducted with CrysAlisPro [Rigaku OD (2017). *CrysAlis PRO*. Rigaku Oxford Diffraction Ltd., Yarnton, Oxfordshire, England]. The data were subjected to analytical absorption correction using CrysAlisPro. The crystal structure was solved using SHELXT [34] and refined on F^2^ by a full-matrix least-squares technique with SHELXL-2016 [34] with anisotropic thermal parameters for all the ordered non-H atoms. In the final refinement cycles, H atoms were repositioned in their calculated positions and treated as riding atoms with 1.5U_eq_(C) for CH_3_. Figure 2 was produced using DIAMOND program [Diamond—Crystal and Molecular Structure Visualization, Crystal Impact—Dr. H. Putz & Dr. K. Brandenburg GbR, Germany, http://www.crystalimpact.com/diamond, accessed on 20 January 2023]. CCDC reference number for compound **2**: **CCDC2215593** (supplementary data available from CCDC, 12 Union Road, Cambridge CB2, 1EZ, UK on request). The crystal data and details of data collection and refinement procedures are collected in Tables S1–S7 in the Supplementary Materials.

### 3.4. Determination of logP by Shake-Flask Method 

The partition coefficient was measured using the methods described earlier [29,30]. An amount of 10 mg of the tested Pt complex **1** or **2** was suspended in 5 mL of 0.9% aqueous NaCl solution, previously saturated with n-octanol, using 10-min sonication. Simultaneously, a similar procedure was carried out using 5 mL of n-octanol pre-saturated with 0.9% NaCl. Both suspensions were then mixed together, shaken for 1 h and centrifuged for 5 min. After phase separation and evaporation under vacuum of a 1 mL sample of each to dryness, the final Pt concentration was determined by ICP-OES (λ = 265.945 nm) and partition coefficients were calculated. For analysis, all samples were dissolved 1:1000 in 2.5% HCl. Each measurement was repeated four times, and the final logP was calculated as the arithmetic mean of the four values. Directly after the experiment, the organic and aqueous phases were additionally tested (TLC) for homogeneity, but no signs of decomposition or isomerization of the tested Pt complexes were found.

### 3.5. Reaction with L-Glutathione

A total of 60 mM solutions of compounds **1** or **2** in dioxane (30 μL) were added to 3 mL of a 2 mM solution of GSH (reduced L-glutathione) pre-saturated with argon in a medium containing 10 mmol/L NaCl and 10 mmol/L Tris xHCl buffer, pH 7.4, reaching a final concentration of 6 µM of the tested complex, and warmed to 37 °C. The progress of the reaction was monitored by UV absorption spectrometry at 260 nm. In this experiment, dioxane was used to dissolve the tested Pt complexes due to its lack of UV absorption at the wavelength used. Argon saturation of GSH was performed to remove oxygen from the solution and thus prevent the formation of S-S bonds that would distort the measurement results.

### 3.6. Cell Culture Used for Testing

The following cancer lines were used for the study: MCF-7 (breast adenocarcinoma), ES-2 (ovarian adenocarcinoma) and A549 (lung adenocarcinoma). Normal cell line BALB/3T3 (murine embryonic fibroblast) was also used for comparison purposes. All these lines were purchased from the American Type Culture Collection (ATCC, Rockville, MD, USA). The preparation of these lines for testing is described in detail in the following paper [22].

### 3.7. Preparation of Stock Solutions of Tested Compounds to In Vitro Studies

The acetone solutions of complexes **1** and **2** were prepared ex tempore by dissolving the complexes in acetone, and the solutions obtained were added to the culture medium (1:9 (*v/v*), respectively) to obtain a stock solution of 1 mg/mL.

### 3.8. In Vitro Cytotoxicity Test through Sulforhodamine B Assay

The test was prepared and performed in the same manner as detailed in the following cited works [22,35,36]. The results are shown in Table 2. 

### 3.9. Total Platinum Uptake Level Test

The survey was conducted exactly according to the method described in the following paper [22]. The results are collected in Table 1.

### 3.10. Statistical Analysis

Statistical analysis was carried out using following software: Statistica (data analysis software system), version 13.3 (2017, TIBCO Software Inc., Palo Alto, CA, USA) and PTC Mathcad Express Prime 6.0.0.0. (Copyright 2019, PTC Inc., Boston, MA, USA). The normal distribution of the data within the groups was verified with the Shapiro–Wilk test and the Kolmogorov–Smirnov (K-S) test with Lilliefors correction. The homogeneity of variances was checked with Levene’s and Brown–Forsythe tests. To avoid a type I error, the statistical significance of differences in the mean IC_50_ values of all of the compounds within a single cell line and in the mean IC_50_ values of each individual compound for cancerous cell lines and healthy reference cells was tested with conservative post hoc tests for multiple comparisons, such as Tukey’s HSD and Bonferroni tests. Moreover, the statistical significance of the differences in the mean IC_50_ values of the tested compounds in relation to the mean IC_50_ values of the reference drug was also tested with Dunnett’s post hoc test. The confidence level (limit) was set as 95% and the threshold for significance level was set at α = 0.05; hence, the differences between the means with a *p*-value (calculated probability value) <0.05 (i.e., at probability level > 95%) were considered statistically significant.

## 4. Conclusions

Studies on the complexation of platinum(II) ions by 4-nitroisoxazoles indicate that this process occurs much more difficultly than in the case of 1-methylnitropyrazoles, where only 1-methyl-3-nitropyrazole did not give the Pt complexes, probably because of the steric hindrance of both methyl and nitro groups that constrain access to lone pairs of pyrazole nitrogen electrons. Furthermore, the electrostatic repelling of the approaching tetrachloroplatinate anion by electron-dense oxygen atoms of the nitro group may also be an important factor that can hinder and/or prevent the formation of an appropriate Pt–nitropyrazole complex [22]. Of the four nitroisoxazoles used, only 3,5-dimethyl-4-nitroisoxazole formed a mixture of isomeric cis and trans complexes with moderate efficiency. In contrast, 4-nitroisoxazole, 3-methyl-4-nitroisoxazole and 5-methyl-4-nitroisoxazole did not yield such complexes. This is probably due to their decomposition under reaction conditions. Many studies on both aromatic systems have shown a lower stability of the aromatic ring of isoxazole than that of pyrazole due, for example, to the much lower delocalization energy of isoxazole and the ease with which the nitrogen–oxygen bond is broken under the influence of nucleophilic (basic) agents, which is due to the bond polarity associated with the difference in electronegativity between nitrogen and oxygen and the longer length than the N-N bond in pyrazole [37,38]. The influence of particularly alkaline factors, especially at elevated temperatures, leads to the easy decomposition of the aromatic ring of isoxazole, so in reactions with isoxazole derivatives it is necessary to use suitably mild conditions, such as in the case of the hydrazinolysis of ester derivatives of isoxazoles [39]. Among isoxazole derivatives, isoxazoles not substituted in position 3 in particular are much less stable [40]. Aromatic rings of isoxazoles unsubstituted in position 3 very readily undergo, even under very mild conditions, decomposition by the breaking of the nitrogen–oxygen bond. This isoxazole ring disruption is particularly favored by an alkaline environment [40]. It is on the decomposition of the isoxazole ring that the activity of the well-known immunosuppressor and anti-inflammatory drug Leflunomide (i.e., 5-methyl-N-[4-(trifluoromethyl) phenyl]-isoxazole-4-carboxamide) is based, where under in vivo conditions the isoxazole ring is easily opened, and its metabolite with a nitrile group is responsible for the actual activity [41]. Therefore, the lack of a positive result for 3-methyl-4-nitroisoxazole in the complexation of Pt(II) is surprising in light of the above, given that this cannot be explained by the steric hindrance of the methyl group in the 3 position, since its derivative, 3,5-dimethyl-4-nitroisoxazole, forms complexes with platinum(II).

Both complexes **1** and **2** showed high cytotoxicity in biological tests, especially compound **2** of trans configuration was surprisingly extremely active, clearly exceeding (more than 15 times) the reference cisplatin against all tested cancer cell lines. *Cis* complex **1** appeared to be more active than cisplatin against MCF7 and A549 cancer cell lines, and comparable to cisplatin against the ES2 cancer cell line. The Pt uptake study showed that the cytotoxicity of compounds **1** and **2** was not due to excessive platination of the tumor cells. Moreover, it was revealed that the platination of cells was significantly lower for the tested compounds than those of cisplatinum, which suggests a molecular mechanism of their biological action. It has been previously shown that the sensitivity of neoplastic tissues to platinum drugs strongly depends on their ability to metabolize and excrete them from the cell [42]. In turn, the rate of metabolism of platinum anticancer drugs and the process of their excretion from the cell is associated with the formation of Pt chelate complexes with endogenous thiols, such as GSH. For this reason, we conducted studies on the reactivity of compounds **1** and **2** to GSH, finding that trans complex **2**, which is more cytotoxic than cisplatin, undergoes this reaction about three times faster, while the compound **1** of cis configuration reacts at a similar rate. This fact, coupled with its high cytotoxicity to normal cells, discouraged us from testing compound **2** for its in vivo antineoplastic activity in a murine tumor model.

The experimental data described in this paper, combined with the conclusions from our previous investigations concerning Pt(II) nitropyrazole complexes, suggest two ways to continue further research in our field of interest. The first is the synthesis of appropriate Pt(II) complexes with various nitroisothiazole ligands [43] in the hope of their greater resistance to GSH while maintaining high cytotoxicity. The second is an attempt at the synthesis and biological research of more stable but usually inactive Pt(IV) complexes with the 3,5-dimethyl-4-nitroisoxazole ligand. This concept is based on the possibility of their bioreductive activation in hypoxic tumor tissues to the trans Pt(II) complex, the high cytotoxicity of which we discovered during the work described in this publication.

## 5. Patents

The results of the research have been patented: Regiec, A.; Mastalarz, H.; Mastalarz, A.; Milczarek, M.; Ryng, St.; Wietrzyk, J.; *Cis*-and *trans*-platinum(II) complex compounds with 3,5-dimethyl-4-nitroisoxazole having antitumor activity, process for their preparation and separation of, Polish patent PL231434B1, 29 February 2019.

## Figures and Tables

**Table 1:** Total cellular platinum uptake in MCF-7 Cells (±SD) and experimentally measured logP ± SD (n = 4) values.

Compound	ng Pt/10^6^ Cells	logP ± SD
**1**(*cis*)	35 ± 8 *	−0.295 ± 0.145
**2**(*trans*)	19 ± 5 *	1.012 ± 0.05
**Cisplatin**	74 ± 20	−2.05 ± 0.17

* The compounds have been absorbed statistically significantly less than cisplatin by MCF-7 cells (for Fisher LSD, Bonferroni, Tukey HSD and Dunnett’s post hoc test, the threshold for significance level was set at α = 0.05. The results were considered statistically significant, when calculated probability of statistical significance had *p*-value < 0.05).

**Table 2:** Cytotoxic activity of platinum derivatives **1–2**, 3,5-dimethyl-4-nitroisoxazole ligand and reference **cisplatin** in normoxia and hypoxia conditions against some cancer and normal cell lines determined in the SRB (sulforhodamine B) viability assay. Data are given as inhibitory concentration IC_50_ that causes a 50% reduction in the cell viability, IC_50_ ± SD [µM].

Compound	Cancer Cells						Normal Cells
	MCF-7		ES-2		A549		BALB/3T3
	Normoxia	Hypoxia	Normoxia	Hypoxia	Normoxia	Hypoxia	Normoxia
**1**(*cis*)	4.19 ± 0.13 **	7.37 ± 2.37 *	6.13 ± 0.71 *	7.37 ± 2.75 *	7.08 ± 0.42 **	9.31 ± 3.12 **	8.31 ± 0.51 *
**2**(*trans*)	0.27 ± 0.16 **	0.78 ± 0.13 **	0.47 ± 0.20 **	0.84 ± 0.07 **	0.60 ±0.13 **	7.7 ± 7.4 **	0.80 ± 0.018 **
**3,5-dimethyl-4-nitroisoxazole**	Inactive	inactive	inactive	inactive	inactive	inactive	>100
**Cisplatin**	12.6 ± 2.6	14.7 ± 5.8	8.6 ± 2.6	13.7 ± 5.7	9.8 ± 1.2	23.9 ± 9.3	8.67 ± 2.6

* Cytotoxicity is statistically significantly comparable to cisplatin, ** statistically significantly more cytotoxic than cisplatin. The underlined values show a statistically significant difference between cytotoxic activity in normoxia and hypoxia conditions. The normal distribution of the data within the groups was verified with the Shapiro–Wilk test and Kolmogorov–Smirnov (K-S) test with Lilliefors correction. The homogeneity of variances was checked with Levene’s and Brown–Forsythe tests. To avoid a type I error, the statistical significance of differences in the mean IC_50_ values of all of the compounds within a single cell line and in the mean IC_50_ values of each individual compound for cancerous cell lines and healthy reference cells was tested with conservative post hoc tests for multiple comparisons, such as Tukey’s HSD and Bonferroni tests. Moreover, the statistical significance of the differences in the mean IC_50_ values of the tested compounds in relation to the mean IC_50_ values of the reference drug was also tested with Dunnett’s post hoc test. The confidence level (limit) was set as 95% and the threshold for significance level was set at α = 0.05; hence, the differences between the means with a *p*-value (calculated probability value) < 0.05 (i.e., at probability level > 95%) were considered statistically significant.

## Data Availability

Not applicable.

## Supplementary Materials

The following supporting information can be downloaded at: https://www.mdpi.com/article/10.3390/molecules28031284/s1.
