# Supplementary material for: Studies on the Complexation of Platinum(II) by Some 4-Nitroisoxazoles and Testing the Cytotoxic Activity of the Resulting Complexes
Source: Molecules. 2023 Jan 28;28(3):1284. doi: 10.3390/molecules28031284 (PMC9920747; doi:10.3390/molecules28031284)
Supplement: Supplementary file 1 [file molecules-28-01284-s001.zip › molecules-2168197-supplementary.pdf]

SUPPLEMENTARY MATERIAL

Studies on the complexation of platinum(II) by some 4-nitroisoxazoles and testing the cytotoxic activity of the resulting complexes

Henryk Mastalarz[1*], Agnieszka Mastalarz[2], Joanna Wietrzyk[3], Magdalena Milczarek[3], Andrzej Kochel[2] and Andrzej Regiec[1*]

[1] Department of Organic Chemistry and Drug Technology, Faculty of Pharmacy, Wrocław Medical University,

211A Borowska Street, 50-556 Wrocław, Poland

[2] Faculty of Chemistry, Wrocław University, 14F Joliot-Curie Street, 50-383 Wrocław, Poland

[3] Hirszfeld Institute of Immunology and Experimental Therapy Polish Academy of Sciences, 12 Rudolf Weigl Street, 53-114 Wrocław, Poland

Table of Contents

Simulated ESI MS spectra of compounds **1** and **2** (**Fig. S1-2**)	p. **2**
Experimental ESI MS spectra of compounds **1** and **2** (**Fig. S3**)	p. **3**
Experimental far IR spectra of compounds **1** and **2 (Fig. S4-5)**	pp **4-5**
Experimental ^{1}H NMR spectra of 3,5-dimethyl-4-nitroisoxazole,compound **1** and **2** (**Fig. S6-8**)	pp 6-7
Crystal data and structure refinement for *trans*-complex **2**	pp 7-8
Atomic coordinates and equivalent isotropic displacement parameters for compound **2** (**Table S2**)	pp 8-9
Bond lengths and angles for *trans*-complex **2 (Table S3)**	pp 10-11
Anisotropic displacement parameters for *trans*-complex **2 (Table S4)**	p. 12
Hydrogen coordinates and isotropic displacement parameters for *trans*-complex **2** (**Table S5**)	p. 13
Torsion angles for *trans*-complex **2** (**Table S6**)	p. 14
Hydrogen bonds parameters for *trans*-complex **2** (**Table S7**)	p. 15

*Corresponding author, e-mail: Henryk Mastalarz (henryk.mastalarz@umw.edu.pl) and Andrzej Regiec (andrzej.regiec@umw.edu.pl)

Simulated (Fig. S1-2) and experimental (Fig. S3) ESI-MS spectra for compound 1 and 2.

Simulation performed with Compass DataAnalysis Software version. 4.2 (copyright 1993-2003 Bruker Daltonik GmbH).

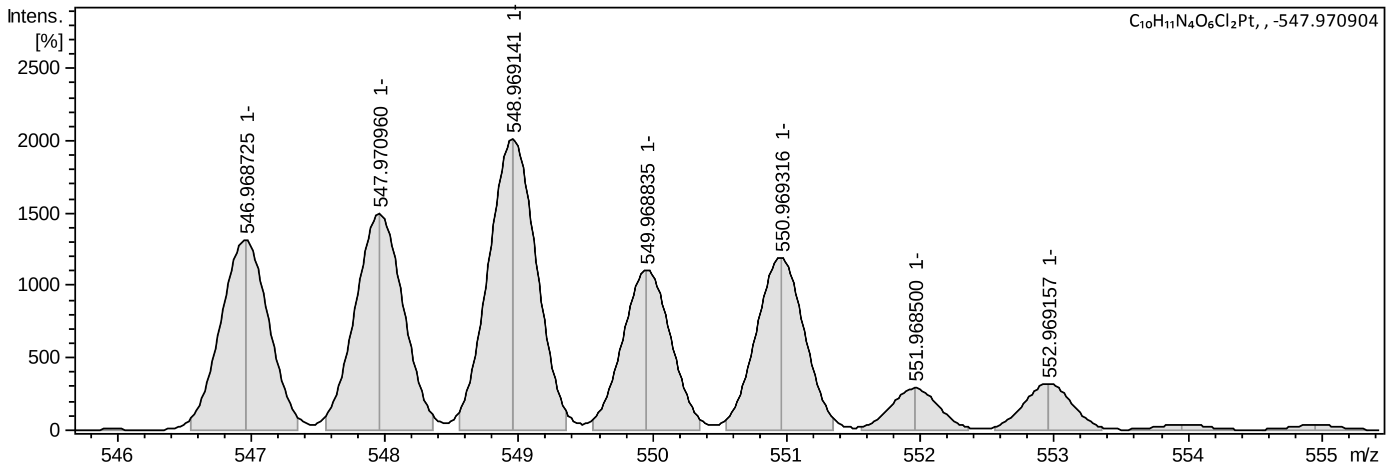

Figure S1. Simulated quasi molecular (547.97096 u/e) and its isotope peaks for formula $[C_{10}H_{12}N_4O_6Cl_2Pt - H]^-$ in ESI-MS (negative ionization) spectrum

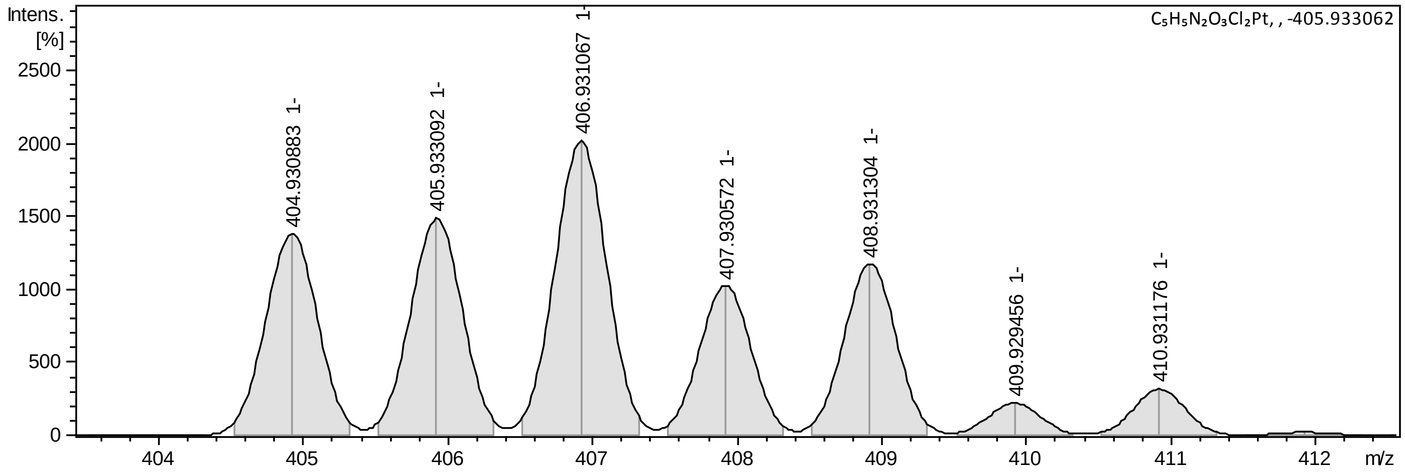

Figure S2. Simulated fragmentation ion peak (406.933092 u/e) and its isotope peaks for formula $[C_5H_6N_2O_3Cl_2Pt - H]^-$ in ESI-MS (negative ionization) spectrum

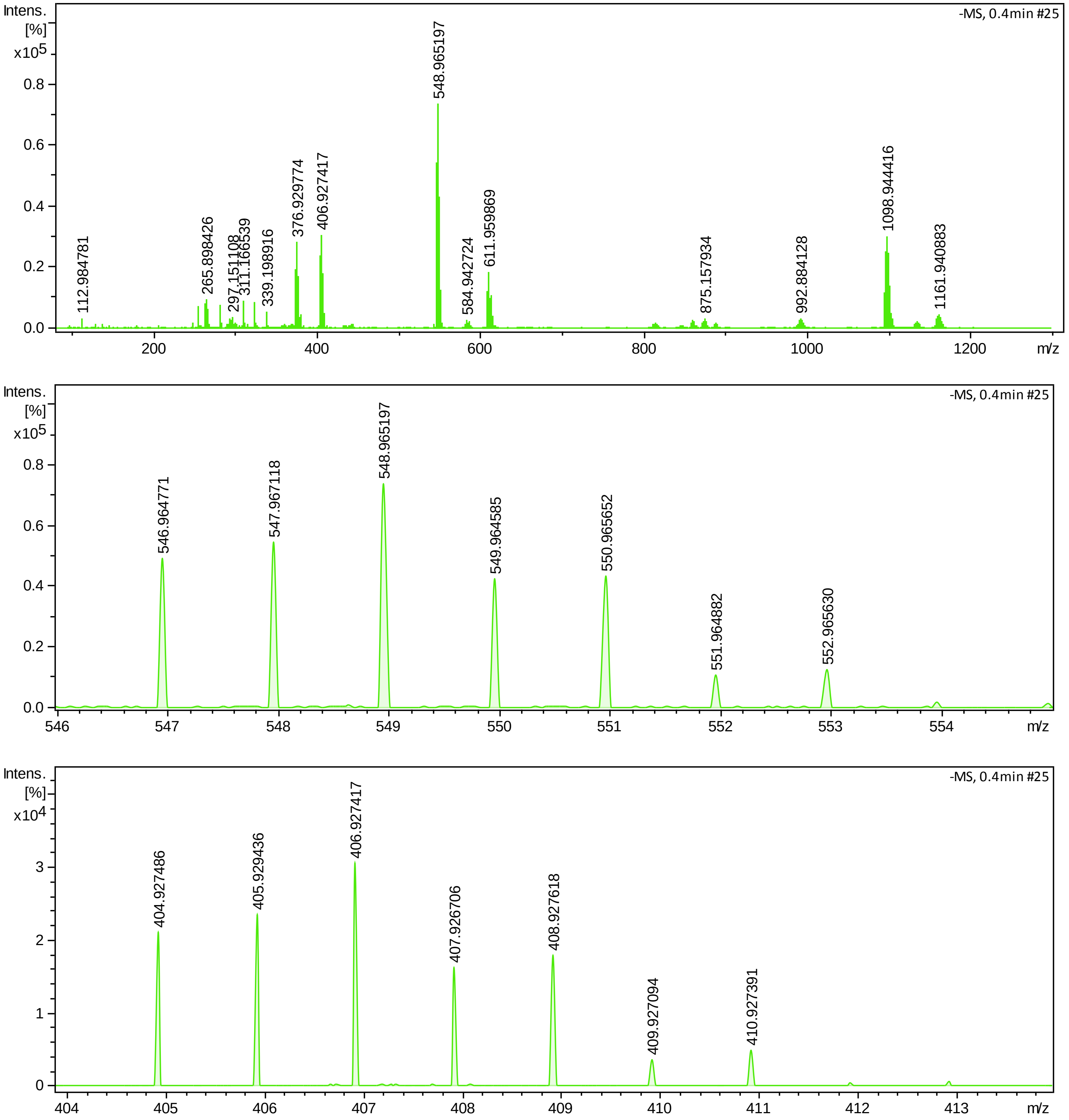

Figure S3. Experimental ESI-MS (negative ionization in MeOH) spectrum of the compounds **1** and **2**. Quasi molecular (parent) and its isotope peaks for formula $[C_{10}H_{12}N_4O_6Cl_2Pt - H]^-$ (547.967118 u/e). Top spectrum – whole spectrum, Middle spectrum- Quasi molecular (parent) and its isotope peaks range (547.967118 u/e). Bottom spectrum - fragmentation ion (405.929436 u/e) and its isotope peaks range.

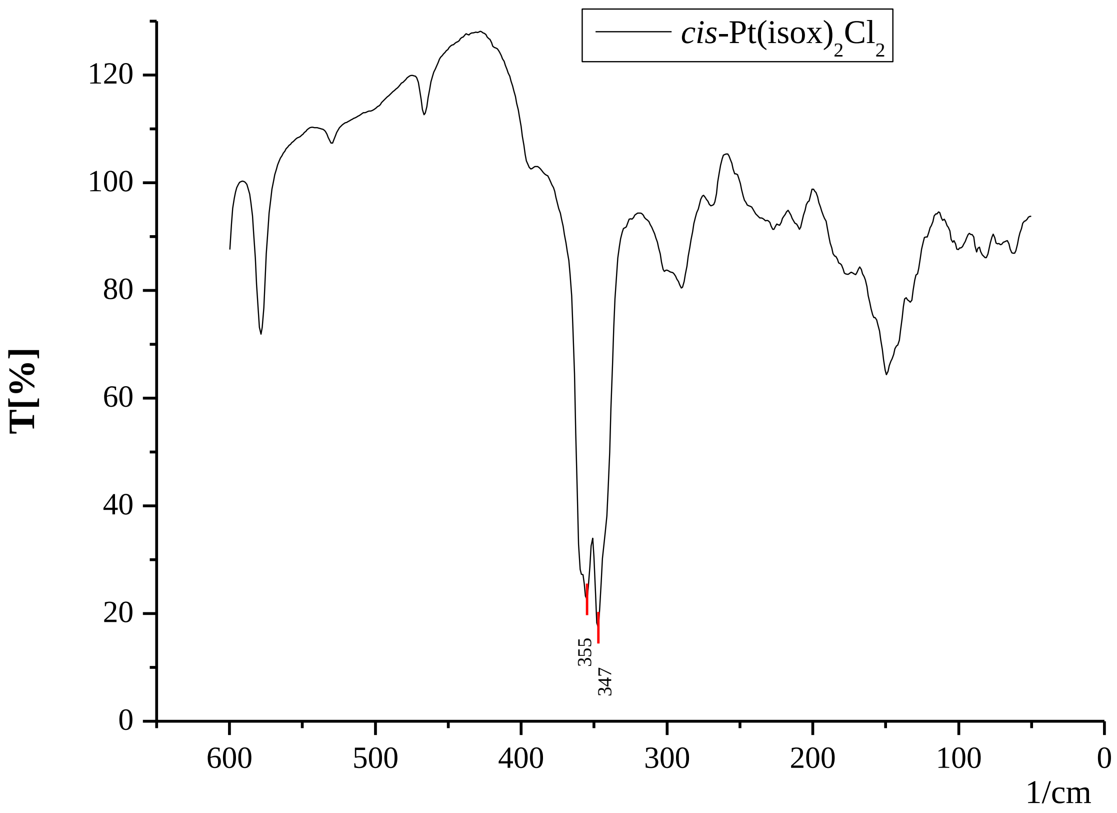

Figure S4. Experimental far IR spectrum (in nujol mull) of the *cis* complex **1**.

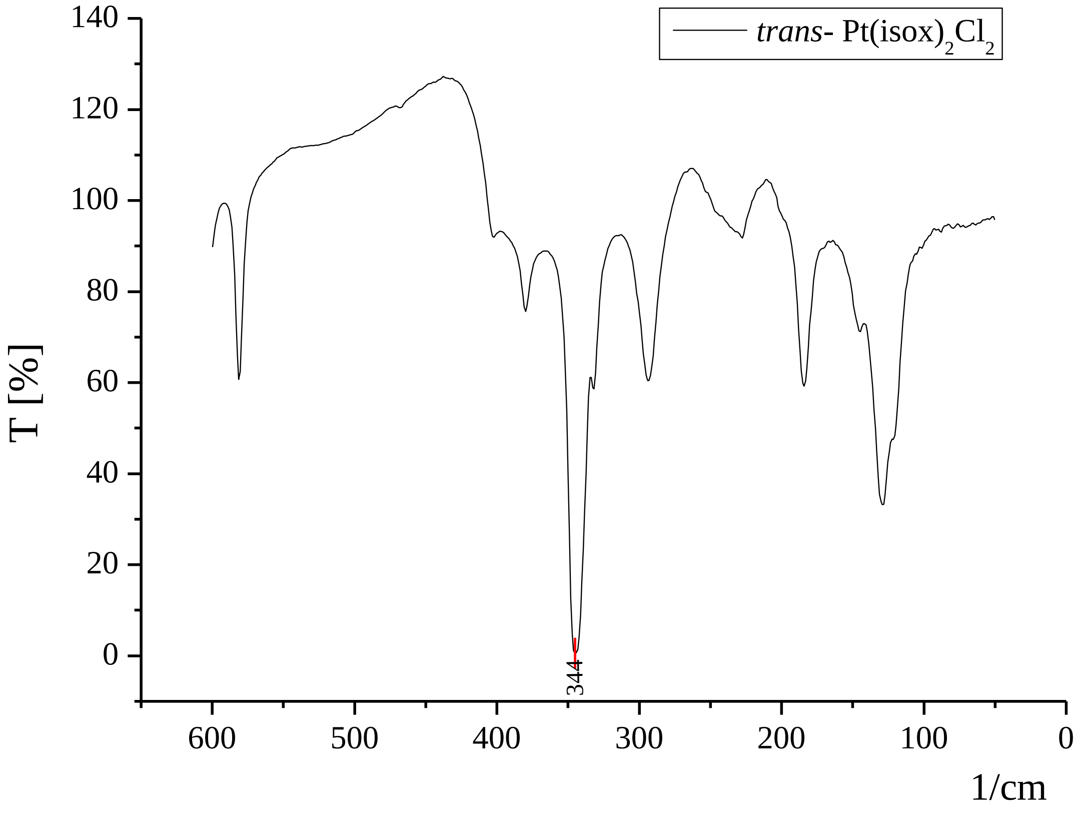

Figure S5. Experimental far IR spectrum (in nujol mull) of the *trans* complex **2**.

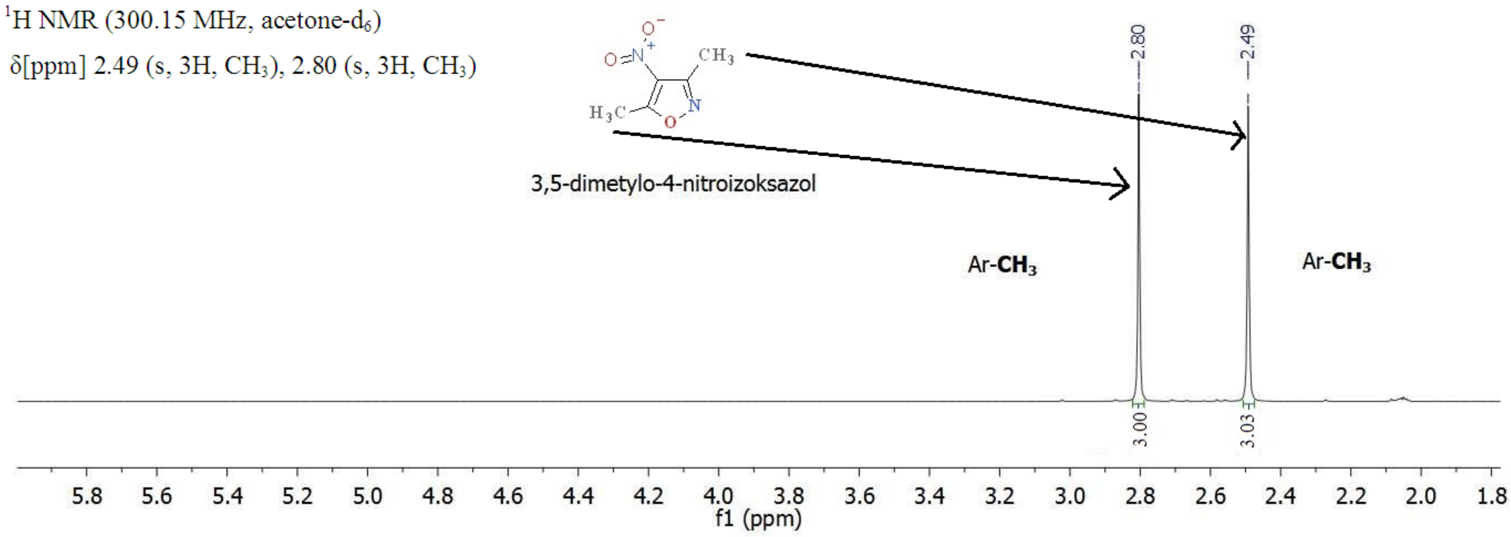

Figure S6. Experimental ^{1}H-NMR spectrum of ligand, i.e.3,5-dimethyl-4-nitroisoxazole. ^{1}H NMR (300.15 MHz, acetone-d_6): δ[ppm] 2.49 (s, 3H, CH_3 in third position of isoxazole ring), 2.80 (s, 3H, CH_3 in fifth position of isoxazole ring).

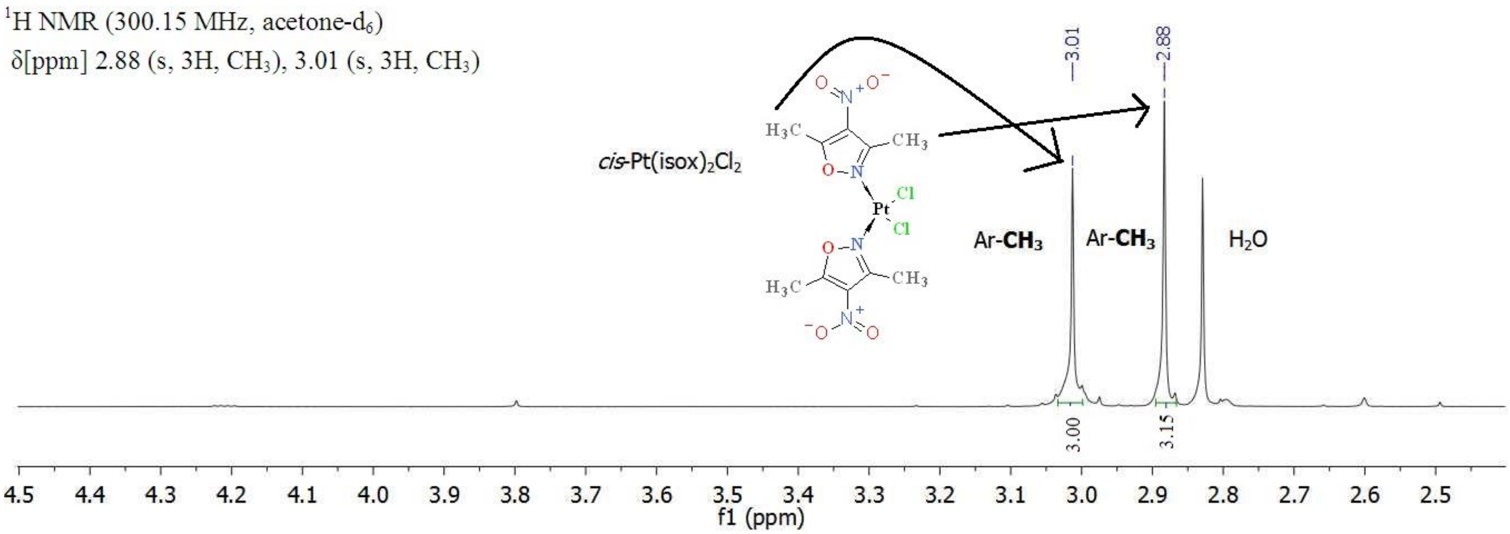

Figure S7. Experimental ^{1}H-NMR spectra of *cis*-complex **1**. ^{1}H NMR (300.15 MHz, acetone-d_6): δ[ppm] 2.88 (s, 3H, CH_3 in third position of isoxazole ring), 3.01 (s, 3H, CH_3 in fifth position of isoxazole ring).

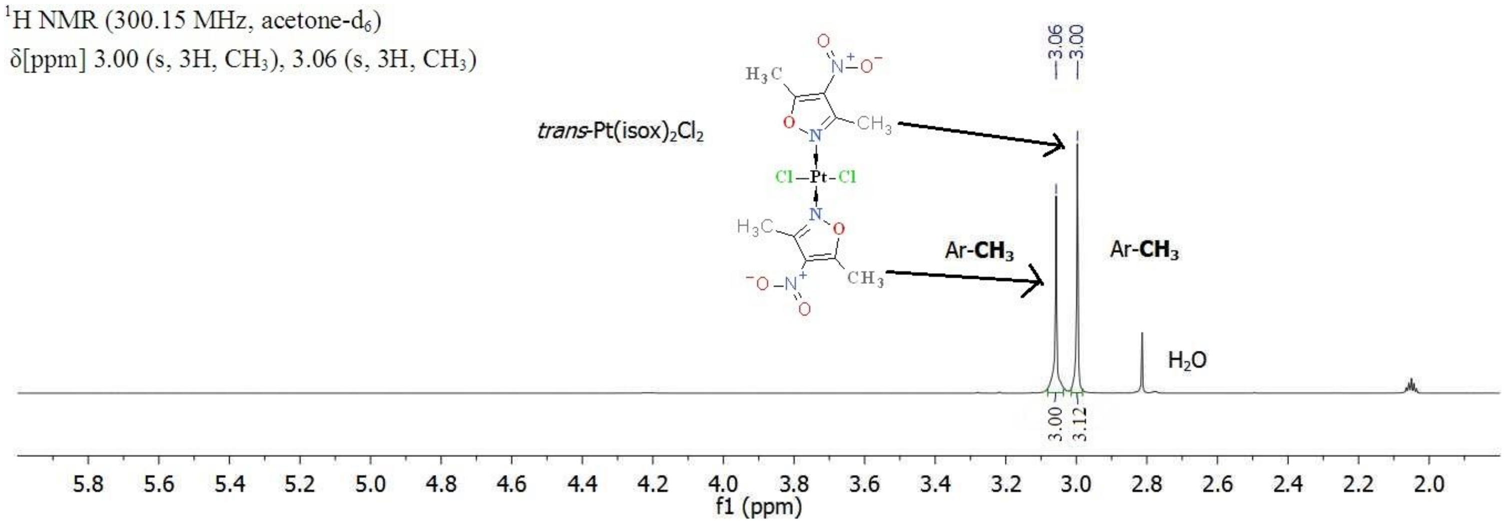

Figure S8. Experimental ^{1}H-NMR spectra *trans*-complex **2**. ^{1}H NMR (300.15 MHz, acetone-d_6): δ[ppm] 3.00 (s, 3H, CH_3 in third position of isoxazole ring), 3.06 (s, 3H, CH_3 in fifth position of isoxazole ring).

Table S1. Crystal data and structure refinement for *trans*-dichlorobis(3,5-dimethyl-4-nitro-isoxazole)platinum(II) (**2**)

**Compound**	**2**
Formula	$C_{10}H_{12}Cl_2N_4O_6Pt$
Formula weight	550.23
Temperature [K]	100(2)
λ [Å]	0.71073
Crystal system	Monoclinic
Space group	P21/n
a [Å]	6.531(3)
b [Å]	8.133(3)
c [Å]	15.419(2)
α [°]	
β [°]	91.86(2)°
γ [°]	
V [$Å^3$]	818.6(5)
Z	2
ϱ_{calc} [Mg m^{-3}]	2.232

Absorption coeff. μ [mm^{-1}]	8.931
F(000)	520
Crystal size [mm]	0.10 x 0.10 x 0.05
θ range[º]	3.350 to 28.802
Index ranges	-8<=h<=8, -10<=k<=10, -20<=l<=20
Reflections collected	17422
Independent reflections	2056 [R(int) = 0.0252]
Completeness to theta = 25.000°	99.9 %
Abs. corr.	analytical
Min., max. transmission factors	1.000 and 0.910
Refinement method	Full-matrix least-squares on F2
Data/restraints/params	2056 / 0 / 108
GOF on F^2	1.095
Final R indices R_1 [I > 2σ(I)]	R1 = 0.0125, wR2 = 0.0254
R indices (all data)	R1 = 0.0158, wR2 = 0.0264
Extinction coefficient	n/a
Largest diff. peak and hole [e $Å^3$]	0.647 and -0.343

Table S2. Atomic coordinates (x 10^4) and equivalent isotropic displacement parameters ($Å^2$x 10^3) for *trans*-complex **2**. U(eq) is defined as one third of the trace of the orthogonalized U^{ij} tensor.

	x	y	z	U(eq)
Pt(1)	5000	5000	5000	12(1)
Cl(1)	6886(1)	6810(1)	4204(1)	21(1)
O(1)	3740(2)	3233(2)	3439(1)	14(1)
N(2)	5264(2)	3265(2)	4103(1)	14(1)
C(3)	6578(3)	2093(2)	3965(1)	15(1)
C(4)	5912(3)	1251(2)	3202(1)	13(1)
C(5)	4187(3)	2009(2)	2890(1)	14(1)
C(6)	8390(3)	1820(3)	4558(1)	25(1)
C(7)	2844(3)	1850(2)	2108(1)	18(1)
N(3)	6957(2)	-62(2)	2788(1)	16(1)

O(2)	5980(2)	-897(2)	2255(1)	21(1)
O(3)	8779(2)	-266(2)	2989(1)	22(1)

Table S3. Bond lengths [Å] and angles [°] for *trans*-complex **2.**

Pt(1)-N(2)#1	1.9874(16)
Pt(1)-N(2)	1.9874(16)
Pt(1)-Cl(1)#1	2.3000(7)
Pt(1)-Cl(1)	2.3000(7)
O(1)-C(5)	1.345(2)
O(1)-N(2)	1.4047(18)
N(2)-C(3)	1.304(2)
C(3)-C(4)	1.417(2)
C(3)-C(6)	1.489(3)
C(4)-C(5)	1.359(3)
C(4)-N(3)	1.429(2)
C(5)-C(7)	1.474(2)
C(6)-H(6A)	0.98
C(6)-H(6B)	0.98
C(6)-H(6C)	0.98
C(7)-H(7A)	0.98
C(7)-H(7B)	0.98
C(7)-H(7C)	0.98
N(3)-O(2)	1.229(2)
N(3)-O(3)	1.231(2)
N(2)#1-Pt(1)-N(2)	180.00(8)
N(2)#1-Pt(1)-Cl(1)#1	91.32(5)
N(2)-Pt(1)-Cl(1)#1	88.68(5)
N(2)#1-Pt(1)-Cl(1)	88.68(5)
N(2)-Pt(1)-Cl(1)	91.32(5)
Cl(1)#1-Pt(1)-Cl(1)	180.000(19)
C(5)-O(1)-N(2)	108.01(13)
C(3)-N(2)-O(1)	108.77(14)
C(3)-N(2)-Pt(1)	134.81(13)
O(1)-N(2)-Pt(1)	116.42(10)
N(2)-C(3)-C(4)	107.62(16)
N(2)-C(3)-C(6)	121.51(17)
C(4)-C(3)-C(6)	130.86(17)
C(5)-C(4)-C(3)	107.64(16)
C(5)-C(4)-N(3)	125.69(17)
C(3)-C(4)-N(3)	126.44(17)

O(1)-C(5)-C(4)	107.91(15)
O(1)-C(5)-C(7)	116.41(16)
C(4)-C(5)-C(7)	135.59(17)
C(3)-C(6)-H(6A)	109.5
C(3)-C(6)-H(6B)	109.5
H(6A)-C(6)-H(6B)	109.5
C(3)-C(6)-H(6C)	109.5
H(6A)-C(6)-H(6C)	109.5
H(6B)-C(6)-H(6C)	109.5
C(5)-C(7)-H(7A)	109.5
C(5)-C(7)-H(7B)	109.5
H(7A)-C(7)-H(7B)	109.5
C(5)-C(7)-H(7C)	109.5
H(7A)-C(7)-H(7C)	109.5
H(7B)-C(7)-H(7C)	109.5
O(2)-N(3)-O(3)	124.81(16)
O(2)-N(3)-C(4)	117.82(16)
O(3)-N(3)-C(4)	117.37(16)

Symmetry transformations used to generate equivalent atoms:

#1 -x+1,-y+1,-z+1

Table S4. Anisotropic displacement parameters ($Å^2 x 10^3$) for *trans*-complex **2**. The anisotropic displacement factor exponent takes the form: $-2\pi^2[h^2 a^{*2}U^{11} + ... + 2 h k a^* b^* U^{12}]$

	U^{11}	U^{22}	U^{33}	U^{23}	U^{13}	U^{12}
Pt(1)	13(1)	13(1)	9(1)	-1(1)	-1(1)	2(1)
Cl(1)	26(1)	20(1)	16(1)	1(1)	3(1)	-4(1)
O(1)	14(1)	16(1)	11(1)	-3(1)	-3(1)	2(1)
N(2)	14(1)	18(1)	10(1)	0(1)	-3(1)	2(1)
C(3)	17(1)	15(1)	12(1)	1(1)	2(1)	2(1)
C(4)	16(1)	12(1)	12(1)	1(1)	4(1)	0(1)
C(5)	16(1)	12(1)	13(1)	-1(1)	4(1)	-3(1)
C(6)	24(1)	31(1)	19(1)	-2(1)	-6(1)	12(1)
C(7)	18(1)	19(1)	17(1)	-5(1)	-2(1)	0(1)
N(3)	18(1)	12(1)	18(1)	2(1)	6(1)	1(1)
O(2)	26(1)	17(1)	19(1)	-5(1)	3(1)	-1(1)
O(3)	16(1)	21(1)	28(1)	0(1)	5(1)	5(1)

Table S5. Hydrogen coordinates (x 10^4) and isotropic displacement parameters ($Å^2$x 10^3) for *trans*-complex **2**.

	x	y	z	U(eq)
H(6A)	8299	2541	5065	37
H(6B)	9645	2070	4252	37
H(6C)	8420	670	4748	37
H(7A)	3543	1205	1670	27
H(7B)	2523	2945	1876	27
H(7C)	1573	1293	2258	27

Table S6. Torsion angles [°] for *trans*-complex **2**.

C(5)-O(1)-N(2)-C(3)	-0.49(18)
C(5)-O(1)-N(2)-Pt(1)	178.97(11)
O(1)-N(2)-C(3)-C(4)	-0.68(19)
Pt(1)-N(2)-C(3)-C(4)	180.00(13)
O(1)-N(2)-C(3)-C(6)	179.54(16)
Pt(1)-N(2)-C(3)-C(6)	0.2(3)
N(2)-C(3)-C(4)-C(5)	1.6(2)
C(6)-C(3)-C(4)-C(5)	-178.6(2)
N(2)-C(3)-C(4)-N(3)	176.33(16)
C(6)-C(3)-C(4)-N(3)	-3.9(3)
N(2)-O(1)-C(5)-C(4)	1.50(18)
N(2)-O(1)-C(5)-C(7)	-175.61(15)
C(3)-C(4)-C(5)-O(1)	-1.9(2)
N(3)-C(4)-C(5)-O(1)	-176.67(16)
C(3)-C(4)-C(5)-C(7)	174.4(2)
N(3)-C(4)-C(5)-C(7)	-0.4(3)
C(5)-C(4)-N(3)-O(2)	-22.2(3)
C(3)-C(4)-N(3)-O(2)	163.95(17)
C(5)-C(4)-N(3)-O(3)	157.36(18)
C(3)-C(4)-N(3)-O(3)	-16.4(3)

Symmetry transformations used to generate equivalent atoms:

#1 -x+1,-y+1,-z+1

Table S7. Hydrogen bonds parameters for *trans*-complex **2** [Å and °].

D-H...A	d(D-H)	d(H...A)	d(D...A)	<(DHA)
Intra C(7)–H(7A)···O(2)	0.98	2.48	3.034(3)	115
C(7)–H(7C)···O(3)^{i}	0.98	2.52	3.477(3)	166

Symmetry code: [i]-*1+x,y,z*
